# Mustard seeds as a safe tool in different application methods for controlling broomrape weed infesting faba bean crop

**DOI:** 10.1186/s12870-026-08948-2

**Published:** 2026-05-19

**Authors:** Mona A. El-Wakeel, Ibrahim M. El-Metwally, Salah A. A. Ahmed

**Affiliations:** Botany Department, Agriculture and Biological Institute, National Research Centre, P.O. 12622, 33 El Bohouth st, Dokki, Giza, Egypt

**Keywords:** Weed control, Broomrape, Faba bean, Mustard, Allelopathy, Intercropping, HPLC

## Abstract

**Background:**

Broomrape is one of the most destructive parasitic weeds limiting faba bean productivity. Mustard has gained attention as a sustainable weed management tool due to its allelopathic potential and richness in bioactive secondary metabolites. Two field experiments were applied in two successive winter seasons to examine different mustard application methods, including foliar spraying, soil incorporation of mustard seed powder and mustard plant intercropped with faba bean crop to identify the most effective strategy for suppressing broomrape infestation and improving faba bean performance. Hence, this study contained ten treatments. Seven of them included different mustard application methods including two interplanting treatments of mustard with faba bean plants in two planting densities. Additionally, two treatments of adding mustard powder beside each faba bean plant (10 and 15 g/plant). Three mustard extract treatments at three successive rates: 15, 30 and 45 g/L. The remaining three treatments included glyphosate–isopropylammonium, Roundup 48% WSC herbicide at 0.18 L ha^− 1^ and two control treatments included hand pulling twice and unweeded check.

**Results:**

Glyphosate is the most effective treatment for controlling broomrape, followed by mustard extract 45, 30 g/L, hand pulling, mustard seed powder at 15 g/plant and mustard Interplanting 3 plants/hill, respectively. In addition, glyphosate herbicide, treatments of mustard extract at 45, 30 g/L, hand pulling, mustard extract 15 g/L as well as mustard seed powder at 15 g/plant, respectively achieved the best results in all faba bean yield and yield traits comparing to other treatments.

**Conclusion:**

Spraying of mustard extract at 45 and 30 g/L was the most effective method of mustard applications to control broomrape as a safe method in comparing with other mustard methods of applications. The bioherbicidal activity of mustard may be attributed to phenolic and flavonoid contents and glucosinolates. HPLC fractionation of mustard seed powder aqueous extract revealed that ferulic, chlorogenic and ellagic acids were the most abundant phenolic acids that may be attributed with the inhibitory herbicidal effect of mustard seed powder.

## Introduction

Faba bean (*Vicia faba* L.) is an annual herbaceous legume and one of the earliest domesticated crops within the Fabaceae family. The seeds are nutritionally valuable with high-quality protein with a well-balanced profile of essential amino acids, dietary fiber and relatively low concentrations of anti-nutritional compounds [1]. The superior protein quality of faba bean has been attributed to the presence of all essential amino acids required for human and animal nutrition [2]. In addition, faba bean is widely cultivated as a rotational crop in Mediterranean agro-ecosystems due to its strong biological nitrogen fixation capacity, which can supply a substantial proportion of crop nitrogen requirements (up to ~ 70%), thereby reducing dependence on synthetic fertilizers [3]. As faba bean is often a weak competitor, effective and well-timed weed management is critical for maintaining stand performance and economic returns. Broomrape (notably *Orobanche crenata* Forsk) represents the most prevalent parasitic weed affecting faba bean cultivation in North and East Africa [4, 5]. This parasite is a non-photosynthetic plant belonging to the Orobanchaceae family, which establishes direct connections with host roots and relies entirely on the host for nutrients and water [6]. Broomrape species are extensively distributed throughout the Mediterranean region and can cause significant yield losses depending on the level of infestation [7]. Severe broomrape (Orobanche spp.) attacks often result in total faba bean crop failure, generating economic damages worth hundreds of millions yearly and endangering livelihoods for about 100 million growers worldwide [8, 9]. The limited success of individual control practices has demonstrated that no single method is sufficient, emphasizing the need for integrated approaches that combine compatible strategies to effectively restrain parasite development and protect crop productivity [5].

Due to increasing restrictions and concerns associated with chemical herbicides, scientific attention has been directed to environmentally safe management options, including biological and integrated control methods [10]. Effective long-term suppression of broomrape relies heavily on depleting its soil seed reservoir, since parasitic seed germination is triggered exclusively by host-derived chemicals [11]. The *Brassicaceae* family is widely recognized for its allelopathic potential in agricultural systems, largely due to the production of glucosinolates and their biologically active secondary metabolites known as allelochemicals that influence germination and growth of neighboring plants. These secondary metabolites, particularly isothiocyanates formed by enzymatic hydrolysis of glucosinolates like sinigrin in mustard tissues, have demonstrated inhibitory effects against a range of weed species and are being explored as sustainable bioherbicide components [12, 13]. In all these regards, the allelopathy phenomenon has been applied as a non-chemical weed management tool in many cropping systems. Notably, the allelopathy phenomenon refers to the release of natural compounds by one plant that can negatively or positively affect the germination or growth of neighboring species.

Mustard (*Sinapis alba* L.*)* within the *Brassicaceae* family seed powder has shown significant suppression of parasitic broomrape [14]. This is attributed to allelopathic active compounds such as fatty acids, glucosinolates, phenolic acids, antioxidants, alkaloids, flavonoids and tannins [15–17]. Additionally, using intercropping has gained traction as a more sustainable weed management approach in weed suppression by capturing light, nutrients and space, thus lowering weed establishment and growth [18]. Intercropping systems involving faba bean with Brassica crops (e.g., mustard) have been widely reported to enhance weed suppression through increased crop competition and allelopathic interactions, improving agroecosystem performance [19]. Despite of the bioherbicidal and allelopathic effects of mustard on annual and parasitic weeds have been previously documented, most intercropping field researches on broomrape control involved non-Brassica intercrops like fenugreek, radish, flax, garlic significantly reduced broomrape infestation levels and seed bank size compared with sole cropping [10, 20, 21]. In all these regards, the novelty of this research lies in applying mustard as an intercrop with faba bean under broomrape-infested conditions, an approach that has not been adequately investigated. In addition, this study examined different mustard application methods, including foliar spraying and soil incorporation of mustard seed powder and mustard plant intercropped with faba bean crop, to identify the most effective strategy for suppressing broomrape infestation and improving faba bean performance. These treatments were compared with conventional control practices, namely hand weeding and glyphosate application, with the objective of identifying safe and sustainable alternatives to chemical herbicides for broomrape management in faba bean production systems.

## Materials and methods

### Study site

Faba bean plants were cultivated over two consecutive winter seasons (2023/2024 and 2024/2025) in a field naturally infested with broomrape at the El Nubaria experimental station of the National Research Centre, Egypt (coordinates 30°31′N, 30°18′E; altitude 21 m). The soil at the site is sandy, and its physical and chemical characteristics, determined using the technique described by Page et al. [22]. The soil at the experimental site was sandy loam in texture, with pH of 8.31 and an electric conductivity (EC) of 0.93 dS/m.Throughout the faba bean growing periods, the average climatic conditions recorded included a daily air temperature of 16–17 °C, relative humidity at 62.0%, wind speed of 2.6–3.5 m/s, precipitation averaging 0.5 mm and solar radiation at 17.0 MJ/m² per day. Maize (*Zea mays* L.) was grown as the prior crop in both seasons.

### Materials

Mustard (*Sinapis alba*) seeds cv. Giza-3 and faba bean (*Vicia faba*) seeds cv. Sakha-1 were purchased from the Agricultural Research Centre, Giza, Egypt. Glyphosate–isopropyl ammonium herbicide was purchased from Shoura company of chemical pesticides.

### Preparation of mustard seed plant material and extract

Mustard seeds were ground to a fine powder using an electric blender. A part gained mustard powder was stored in plastic bags to be applied directly in the field. The remained amount of seed powder was used to prepare a stock solution (50% w/v) of mustard seeds’ aqueous extract. In a 4 L Erlenmeyer flask, 1000 g of ground mustard powder was placed and 2 L of distilled water was added. The shaker (200 revolutions per minute) was used to shake the beaker at room temperature for 48 h. To remove debris, the mixture was filtered through four layers of cheesecloth and centrifuged for 30 min. After that the supernatant was filtered through one layer of filter paper (Whatman No. 1). Following filtration, mustard aqueous extracts at successive concentrations of 15, 30 and 45% (w/v) were prepared from the prepared 50% stock solution. The extraction method was repeated as needed to ensure that the extracts were always fresh.

### Treatments and design

Land was divided in a completely randomized block arrangement in four replicates into 40 plots with a net size of 10.5 m^2^ for each, comprising five furrows 3.5 m in length and 0.60 m in width. In November (the 20th and 13th in the first and second seasons, respectively) in both seasons, faba bean seeds cv. Sakha-1 were sown (2 seeds per hill), with 0.25 m space on the two sides of the ridge. Ten treatments were applied in a random complete block design with four replicates. Seven treatments of them included different mustard application methods that were applied. Two interplanting treatments that mustard seeds were drilled between the two sides of faba bean in two planting densities (2 and 3 mustard plants/hill). Additionally, two treatments of adding mustard powder beside each faba bean plant (10 and 15 g/plant). Three mustard extract treatments at three successive rates, 15, 30 and 45 g/L. The remained three treatments included glyphosate–isopropylammonium, Roundup 48% WSC (isopropylammonium N-(phosphonomethyl) glycinate) herbicide at 0.18 L ha^− 1^ and two control treatments included hand pulling twice and unweeded check. Two control treatments included hand pulling twice and unweeded check. Mustard powder and extracts, as well as hand pulling twice and glyphosate herbicide were added during faba bean flowering stages (60 and 75 DAS).

All treatments at 25 DAS, faba bean plants were hand pulled from other annual weeds and thinned, leaving one plant per hill. During growth stages, plants were irrigated through a drip irrigation system using emitters of 2.0 L h⁻¹ capacity. At 70 DAS, mustard plants were removed to avoid the competition with faba bean plants. Also, all treatments were fertilized with ordinary single superphosphate (15.5% P₂O₅) at 360 kg ha⁻¹ during land preparation. All experimental plots received ammonium nitrate (33.5% N) at a rate of 96 kg ha⁻¹ divided into four equal addition doses at 20, 30, 40 and 50 DAS. During growth stages, plants were irrigated through a drip irrigation system using emitters of 2.0 L h⁻¹ capacity. 

### Metrics

#### Broomrape traits

At harvest (on April 30th and April 12th in the first and second seasons, respectively) broomrape weeds were uprooted for estimating the number, total fresh biomass and dry weight per m².

#### Faba bean traits

##### Growth traits

At 110 DAS, plant height, leaves number, branches number, SPAD value [23] and fresh as well as dry weight of stem, leaves and whole plant.

##### Yield traits

At maturity, 10 faba bean plants were randomly uprooted from each plot to estimate plant height (cm). Moreover, number of pods/plant, weight of pods/plant, pod length (cm), number of seeds/pod, weight of 100 seeds (g), biological yield (g/plant) and seed yield (ardab/fed). 

##### Faba bean seed quality

**Chemical composition**

Seeds from each treatment were collected, cleaned and crushed to tiny powder using a coffee grinder and prepared for analysis.

a Total protein
  Total nitrogen (N%) content was determined by the micro-Kjeldahl method described by AOAC [24]. Total protein content was calculated by multiplying the value of total nitrogen by 6.25.
  $${\mathrm{TP}}\left(\%\right)={\mathrm{TN}}\left(\%\right)\times6.25$$
b According to Hedley et al. [25], total carbohydrates in the faba bean seeds were estimated. The absorbance was read with a JASCO V-750 spectrophotometer at 490 nm based on a standard curve of glucose against a blank and then expressed as mg/g DW.

#### Estimation of mustard active secondary metabolites

##### Quantitative estimation of total secondary metabolites in mustard

a Total glucosinolates (µmol/g DW)
  Glucosinolates were extracted from the dried seed powder of mustard. Their content was estimated by measuring the amount of glucose released after enzymatic hydrolysis by the myrosinase enzyme (Rauchberger et al. [26]). The liberated glucose was then quantified using a colorimetric method as described by Nasirullah [27].
b Total phenolic content (mg/g DW)
  The total phenolic compounds in mustard seeds were assessed calorimetrically using the Folin–Ciocalteu reagent following the procedure outlined by Fu et al. [28].

##### HPLC instrumentation for qualitative determination of phenolic acids

High-performance liquid chromatography (HPLC) was used to fractionate phenolic acids in mustard aqueous extract according to the procedure described by Kim et al. [29]. HPLC analysis was carried out using an Agilent 1260 series. The separation was carried out using a Zorbax Eclipse Plus C8 column (4.6 mm x 250 mm i.d., 5 μm). The mobile phase consisted of water (A) and 0.05% trifluoroacetic acid in acetonitrile (B) at a flow rate of 0.9 ml/min. The mobile phase was programmed consecutively in a linear gradient as follows: 0 min (82% A); 0–1 min (82% A); 1–11 min (75% A); 11–18 min (60% A); 18–22 min (82% A); 22–24 min (82% A). The multi-wavelength detector was monitored at 280 nm. The injection volume was 5 µl for each of the sample solutions. The column temperature was maintained at 40 °C. Phenolic acids identification was based on matching retention times and UV spectra with those of authentic standards.

### Statistical analysis

The collected data were subjected to a homogeneity test prior to analysis of variance (ANOVA). The outputs proved that the homogeneity and normality of the data are satisfied for running further ANOVA. Thus, data of each season were subjected to ANOVA according to Casella [30], using the Costat software program, Version 6.303, 2004. Seasons. The means of applied treatments were separated using Duncan’s multiple range test (alphabetical lowercase letters) at the 0.05 level of probability.

## Results

### Weed growth parameters

**Fig. 1:**
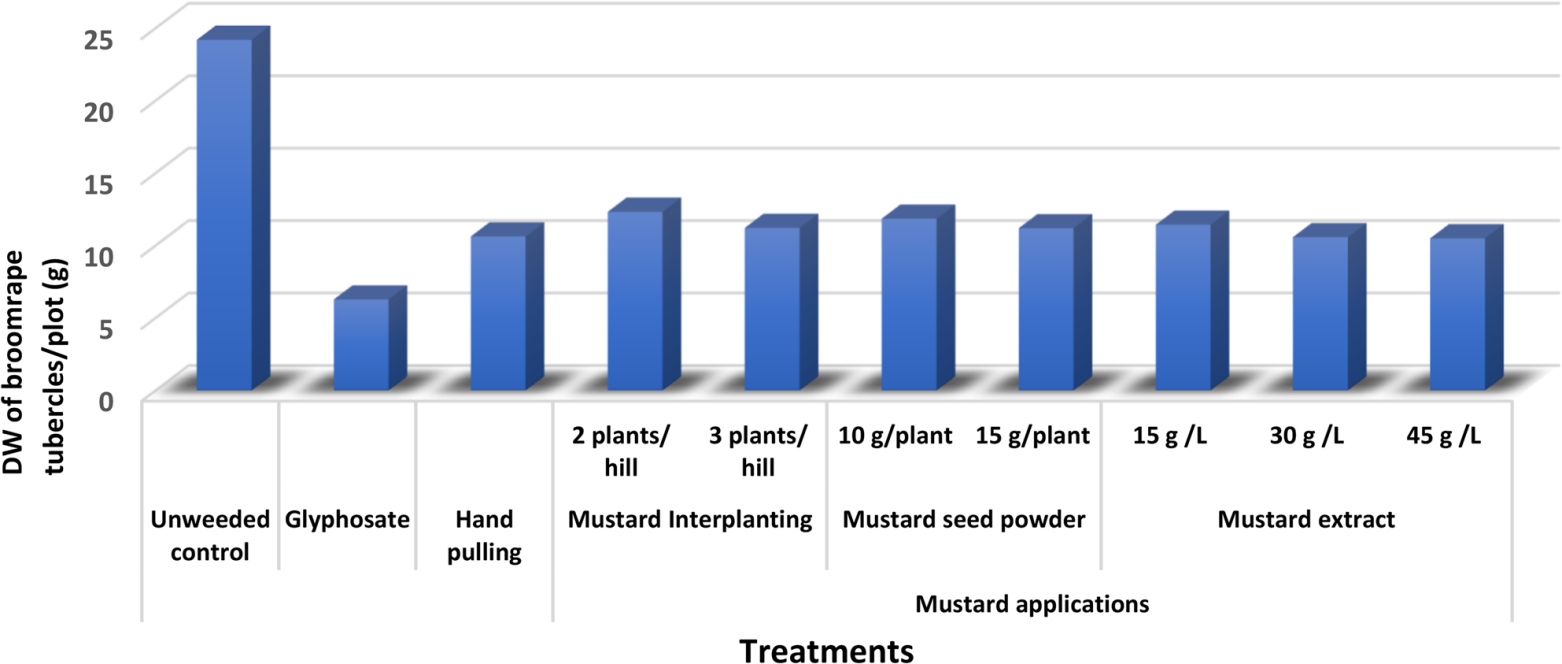
Mean comparison of broomrape dry weight at harvest as influenced by broomrape management treatments (combined analysis of two seasons)

The results in Table 1 revealed that all different types of mustard application and herbicidal glyphosate treatment significantly suppressed broomrape infested faba bean plants and decreased the number, fresh and dry weight of broomrape tubercles/ plot at harvest as compared to their corresponding infected control. Glyphosate is the most effective treatment for controlling broomrape, followed by mustard extract 45, 30 g/L, hand pulling, mustard seed powder at 15 g/plant and mustard Interplanting 3 plants/hill, respectively. These treatments decreased the dry weight of broomrape tubercles/plots by 74.08, 56.63, 56.30, 56.10, 53.74 and 53.66%, respectively when compared to their corresponding infected control. The broomrape control efficiencies of all applied weeded methods are depicted in Fig. 1.

**Table 1:** The impact of different types of mustard application and glyphosate herbicide on broomrape growth parameters/m² infesting in faba bean at harvest stage (combined analysis of two seasons)

Treatments		∗No. of broomrape tubercles/m²	∗F.W. of broomrape tubercles/m² (g)	∗D.W. of broomrape tubercles/m² (g)
Mustard Interplanting	2 plants/hill	12.4f	53.70f	12.32e
	3 plants/hill	11.2d	48.00d	11.21bcd
Mustard seed powder	10 g/plant	11.9ef	51.60ef	11.85de
	15 g/plant	11.1d	47.10d	11.19bcd
Mustard extract	15 g /L	11.5de	49.20de	11.43 cd
	30 g /L	10.1c	43.77c	10.57b
	45 g /L	9.3b	40.30b	10.49b
Hand pulling		10.4c	43.96c	10.62bc
Glyphosate		5.4a	23.40a	6.27a
Unweeded control		25.2 g	108.77 g	24.19f
LSD	5%	0.6	2.79	0.86

*No. number, *FW* fresh weight, *DW* dry weight

### Faba bean traits

#### Growth traits

All growth parameters of faba bean plants, represented by plant height (cm), number of leaves/plant, and number of branches/plant, were significantly increased by the investigated broomrape at 110 DAS, as compared to the corresponding infected control (Table 2). The maximum high significant increases in these mentioned growth parameters were obtained with glyphosate herbicide followed by mustard extract at 45, 30 g/L, hand pulling, mustard extract 15 g/L, as well as mustard seed powder at 15 g/plant, recording an increase in plant height by 44.95, 43.27, 38.69, 37.00, 35.93 and 31.35%, respectively over the unweeded control. Notably, growth parameters enhancement response was concentration dependent for either mustard seed powder or extract used. With regard to SPAD value, results in Table 2 also showed that all applied methods increased this character except mustard extract at 15 g/L compared to the weedy check. Treatments of Interplanting 3 plants/hill, mustard extract at 45 g/L, mustard seed powder at 15 g/plant, hand pulling, interplanting 2 plants/hill, and mustard seed powder at 10 g/plant recorded the highest values of SPAD as compared to the corresponding infected control. On the contrary, mustard extract at 15 g/L recorded the lowest value of SPAD.

**Table 2:** Mean comparison of faba bean growth parameters at 110 DAS as influenced by different broomrape management treatments under broomrape infection (combined analysis of two seasons)

Treatments		Plant height (cm)	∗No. of leaves / plant	∗No. of branches/plant	SPAD value
Mustard Interplanting	2 plants/hill	75.4 g	25.23i	3.2e	41.90ab
	3 plants/hill	77.3f	36.30 h	3.3e	43.23a
Mustard seed powder	10 g/plant	83.7e	38.10 g	3.7d	40.83bc
	15 g/plant	85.9d	39.00f	3.7d	42.33ab
Mustard extract	15 g/L	88.9c	41.57e	4.0c	35.40e
	30 g/L	90.7b	44.13c	4.0c	39.20d
	45 g/L	93.7a	46.20b	4.6b	42.50a
Hand pulling		89.6bc	43.25d	4.0c	41.96ab
Glyphosate		94.8a	47.13a	5.0a	40.13d
Unweeded control		65.4 h	22.37j	2.7f	35.93e
LSD at 5%		1.5	0.87	0.2	1.55

*No. number

The growth traits result of faba bean i.e., fresh weight of leaves/plant, fresh weight of stem, fresh weight of plant, dry weight of leaves/plant, dry weight of stem and dry weight of plant were highly significantly increased as affected by all applications of different types of mustard and glyphosate herbicide treatments comparing to their corresponding control (Table 3). Thus, glyphosate herbicide showed the maximum values of all growth traits of faba bean followed by mustard extract at 45, 30 g/L, hand pulling, mustard extract 15 g/L as well as mustard seed powder at 15 g/plant, respectively, compared to other treatments. The increases in dry weight of faba bean plant as affected by these treatments reached to 112.25, 86.57, 64.08, 59.47, 53.25 and 47.93%, respectively when compared to the unweeded control.

**Table 3:** Mean comparison of faba bean growth parameters at 110 DAS as influenced by different broomrape management treatments under broomrape infection (combined analysis of two seasons)

Treatments		Fresh weight of			Dry weight of		
		Leaves/plant (g)	Stem (g)	plant(g)	Leaves/plant (g)	Stem (g)	plant(g)
Mustard Interplanting	2plants/hill	82.33e	115.13 h	197.46i	10.10 h	11.60e	21.70 g
	3plants/hill	87.37d	119.43 g	206.80 g	11.20 g	12.50e	23.70f
Mustard seed powder	10 g/plant	83.30e	119.17 g	202.47 h	11.00 g	12.40e	23.40f
	15 g/plant	88.53d	122.23f	210.76f	11.50ef	13.50 cd	25.00e
Mustard extract	15 g/L	93.73c	133.57e	227.30e	11.90e	14.00c	25.90de
	30 g/L	94.27c	139.27c	233.54c	13.40c	14.33c	27.73c
	45 g/L	104.33b	144.37b	248.70b	15.20b	16.33b	31.53b
Hand pulling		94.05c	136.45d	230.50d	12.70d	14.25c	26.95 cd
Glyphosate		138.23a	150.60a	288.83a	17.27a	18.60a	35.87a
Unweeded control		75.70f	79.53i	155.23j	7.77i	9.13f	16.90 h
LSD at 5%		1.43	1.86	1.78	0.42	1.03	1.25

#### Yields trait

**Fig. 2:**
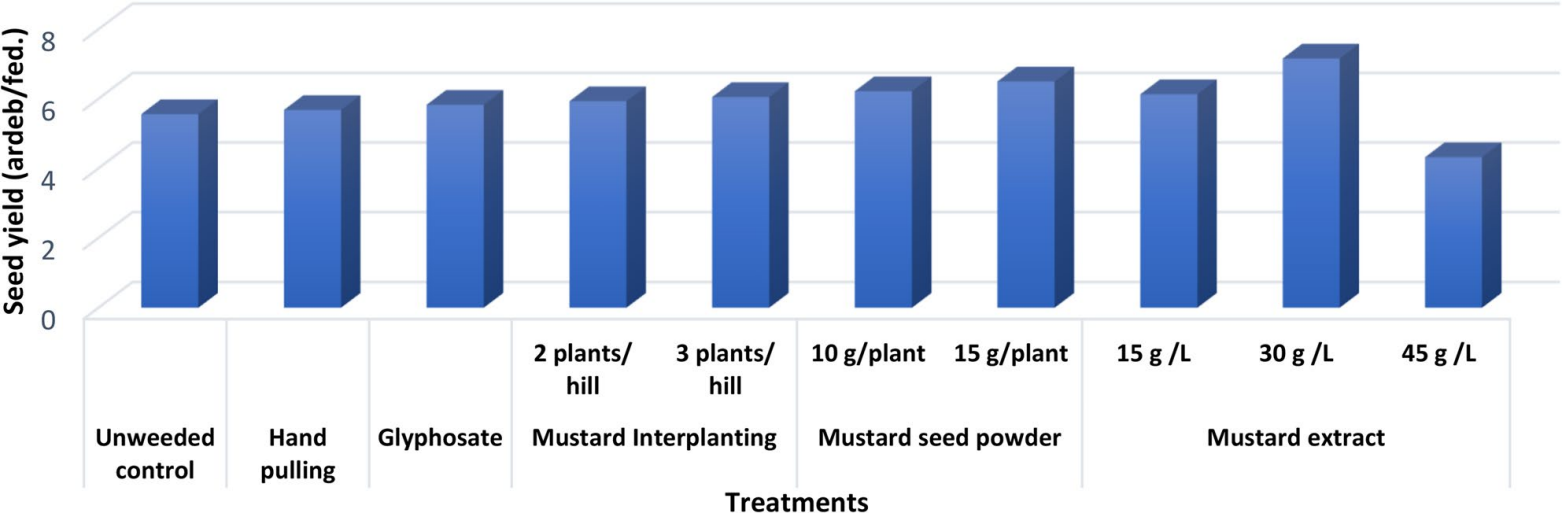
Seed yield (ardeb/fed.) as affected by different broomrape management treatments under broomrape infection (combined analysis of two seasons)

Faba bean yield and yield attributes such as plant height, number of pods/plant, weight of pods/plant (g), pod length (cm), number of seeds/pod, weight of 100 seeds (g), bioyield, and yield of seeds revealed highly significant increases as compared to their corresponding control (Table 4). Maximum increases in these parameters were recorded by glyphosate herbicide over the untreated control. In addition, treatments of mustard extract at 45, 30 g/L, hand pulling, mustard extract at 15 g/L, as well as mustard seed powder at 15 g/plant, respectively achieved the best results in all faba bean yield and yield traits comparing to other treatments. These treatments increased the weight of 100 seeds (g) by 42.55, 32.62, 29.80, 29.08, 26.98 and 25.55%, respectively and increased the yield of seeds by 65.74, 50.46, 43.98, 41.90, 40.05 and 37.27%, respectively when compared to their unweeded control (Fig. 2).

**Table 4:** Yield and yield attributes of faba bean at harvest as affected by different broomrape management treatments under broomrape infection (combined analysis of two seasons)

Treatments		Plant height (cm)	Yield and yield attributes parameters						
			∗No. of pods/plant	∗Wt. of pods/plant	Pod length (cm)	∗No. of seeds/pod	∗Wt. of 100 seeds (g)	Bio yield (g/plant)	Seed yield (ardab/fed.)
Mustard Interplanting	2 plants/hill	81.6 h	10.7e	29.40f	9.1 cd	4.2c	65.77e	91.20f	5.56 g
	3 plants/hill	86.3f	11.0de	29.90ef	9.3bcd	4.3c	66.77e	92.80e	5.68 g
Mustard seed powder	10 g/plant	84.3 g	11.0de	30.20ef	9.7abc	4.4c	67.57e	93.00e	5.83efg
	15 g/plant	89.1e	11.5cde	31.30def	10.5abc	4.6b	69.93d	93.70e	5.93def
Mustard extract	15 g/L	89.2e	11.7 cd	31.80cde	10.7abc	5.1b	70.73 cd	95.37d	6.05cde
	30 g/L	92.7c	12.3bc	33.80bc	11.3abc	5.3b	72.30bc	96.67bc	6.22bc
	45 g/L	98.4b	13.0b	35.70b	11.5ab	5.5ab	73.87b	97.37b	6.50b
Hand pulling		91.5d	11.9c	32.60 cd	11.0abc	5.2b	71.90c	96.15 cd	6.13 cd
Glyphosate		99.5a	15.7a	43.03a	11.9a	5.9a	79.70a	105.20a	7.16a
Unweeded control		78.1i	6.3f	17.30 g	7.3d	3.0d	55.70f	65.57 g	4.32 h
LSD at 5%		1.1	0.9	2.3	2.3	0.5	1.85	1.22	0.29

*No. number, *Wt*. weight

#### Faba bean seed quality

a Protein percentage:
  The results recorded in Table 5 indicated that protein percentage in faba bean seeds was significantly increased by hand pulling (29.85%), followed by mustard Interplanting (3 plants/hill) (28.49%) and mustard extract at 45 g/L (27.43%) and mustard seed powder at 10 g/plant (25.56%). These superior treatments increased the average of protein percentage more than the unweeded treatment.
b Carbohydrates:
  The tabulated res ults in Table 5 revealed that glyphosate herbicide followed by mustard interplanting 3 plants/hill, mustard Interplanting 2 plants/hill, mustard seed powder at 10 g/plant and mustard extract at 15 g/L caused significant increases in carbohydrates over the untreated control.

**Table 5:** Faba bean seed quality as affected by different broomrape management treatments under broomrape infection (combined analysis of two seasons)

Treatments		% of Protein	Carbohydrates (ppm)
Mustard Interplanting	2 plants/hill	21.15 g	170.234c
	3 plants/hill	28.49b	179.119b
Mustard seed powder	10 g/plant	25.56d	157.156d
	15 g/plant	20.81 g	117.730i
Mustard extract	15 g/L	23.89e	154.046e
	30 g/L	21.82f	129.731 g
	45 g/L	27.43c	103.550j
Hand pulling		29.85a	126.355 h
Glyphosate		21.12 g	188.051a
Unweeded control		25.24d	148.999f
LSD at 5%		0.52	1.845

### Estimation of mustard active secondary metabolites

#### Quantitative estimation of total secondary metabolites in mustard

Chemical analysis of mustard seed water extract revealed that the total content of glucosinolates was 288.59 µmol/g dry weight. However, total estimated phenolic contents were 43.6 mg/g dry weight of mustard seed powder.

#### HPLC instrumentation for qualitative determination of phenolic acids

**Fig. 3:**
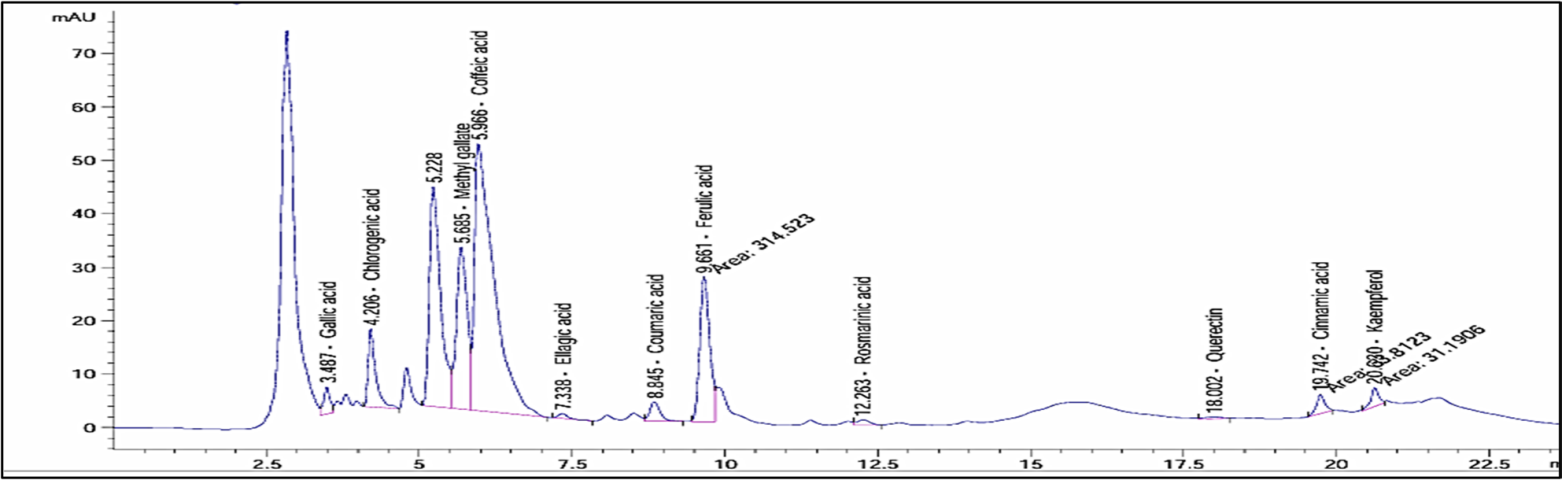
HPLC fractionation of phenolic acids in mustard seed powder water extract

Table 6 presents the polyphenolic compounds determined by HPLC in mustard water extract. It was shown that ferulic, chlorogenic and ellagic acids are the major compounds found in the extract. Additionally, coffeic, methyl gallate, and ferulic acid were the most abundant phenolic acids in the mustard seed powder water extract. Moreover, other phenolic acids are present in minor concentrations as shown in Fig. 3.

**Table 6:** HPLC phenolic acids fractionation in mustard seed water extract expressed as µg/ml

Phenolic acids	Area	Conc. (µg/ml)
Gallic acid	36.54	2.65
Chlorogenic acid	128.09	17.95
Catechin	0.00	0.00
Methyl gallate	375.74	21.30
Caffeic acid	1120.14	67.06
Syringic acid	0.00	0.00
Rutin	0.00	0.00
Ellagic acid	10.18	1.16
Coumaric acid	42.83	1.56
Vanillin	0.00	0.00
Ferulic acid	314.52	18.19
Naringenin	0.00	0.00
Rosmarinic acid	13.85	1.23
Daidzein	0.00	0.00
Quercetin	7.26	0.94
Cinnamic acid	33.81	0.73
Kaempferol	31.19	3.20
Hesperetin	0.00	0.00

## Discussion

Overall, the findings indicated that yellow mustard (*Sinapis alba* L.) seed powder and its aqueous extracts contain biologically active phenolic acids with herbicidal potential. Therefore, they may represent safe alternatives to chemical herbicides for broomrape control [14]. Comprehensive literature reviews showed that these phenolic acids act by multiple mechanisms, e.g., altering membrane permeability, disrupting cell division, inhibiting enzyme activity, and interfering with hormone signaling, supporting their allelopathic and phytotoxic roles [31]. Torrijos et al. [32] concluded that yellow mustard exhibited comparatively high levels of phenolic acids, including sinapic, p-hydroxybenzoic, and ferulic acids, along with several flavonoids such as luteolin and quercetin that were characteristic of this seed fraction. Accordingly, HPLC analysis of the aqueous extract in the present study identified several bioactive phenolic compounds, including ferulic, chlorogenic, caffeic, ellagic, coumaric, cinnamic, and rosmarinic acids, in addition to flavonoids such as quercetin and kaempferol. These compounds have been widely reported to possess allelopathic and phytotoxic activities that may inhibit weed seed germination and interfere with physiological processes [33–35]. In addition to these phenolic constituents, up to five glucosinolates were detected, among them sinigrin, sinalbin, progoitrin and glucobrassicin. Sinigrin and its precursor breakdown products formed through myrosinase-mediated hydrolysis were also identified. In mustard, sinalbin represents the dominant glucosinolate and is enzymatically converted into p-hydroxybenzyl isothiocyanate, a non-volatile antimicrobial compound with limited stability. The instability of this compound likely accounts for its absence in earlier analytical assessments [36, 37]. Depending on the allelopathic potential of mustard plant through releasing allelochemicals to the surrounding rhizosphere, light, nutrient and water competition, the intercropping of mustard plant with faba bean plants suppressed broomrape germination and growth and scored as an effective management tool than faba bean monoculture [10, 21, 32]. Therefore, the biological responses observed following the application of mustard seed powder and its aqueous extract can be explained by the combined biochemical behavior of these secondary metabolites, rather than that of a single compound alone. Although, only a few reports were focused on these yellow mustard seed bioactive compounds’ mode of action on broomrape parasitic weed, the observed growth-inhibitory effects highlighted the potential of mustard-based treatments as environmentally friendly alternatives for sustainable weed and parasitic plant management.

Further studies evaluated glyphosate as a post-emergence systemic herbicide that improved the capability of controlling parasitic broomrape species. Glyphosate interferes with the production of aromatic amino acids by blocking the activity of the enzyme 5-enolpyruvylshikimate-3-phosphate synthase (EPSPS). In contrast, herbicides belonging to the imidazolinone and sulfonylurea groups act by inhibiting acetolactate synthase (ALS), also known as acetohydroxyacid synthase (AHAS), which is essential for the synthesis of branched-chain amino acids, including isoleucine, leucine and valine [38]. This herbicide reflects a systemic mode of action, as it is taken up by both leaves and roots and is efficiently translocated within the plant resulting in effective suppression of the parasite [39]. Moreover, hand pulling has several limitations as a safe control method for parasitic broomrape. In many cases, the parasite causes damage to the host plant before it emerges above the soil surface. Early manual removal may also lead to partial uprooting or injury of the crop, since the parasite is closely attached to the host roots. In addition, new parasite shoots can appear shortly after removal. This method becomes less practical when weed infestation is severe. Furthermore, the removed shoots must be collected and destroyed immediately to prevent reinfestation. Furthermore, hand pulling usually does not increase crop yield in the same growing season, but it was proven to reduce the parasite seed bank in the soil over time [40].

As different mustard methods suppressed the parasitic weed, it promoted faba bean productivity. This suggests that the presence of bioactive allelochemicals such as glucosinolates and phenolics may disrupt broomrape germination and attachment mechanisms while freeing the host from parasitic competition, enabling improved growth and reproductive output of faba bean crop or the selective stimulating response of faba bean plants enhanced its growth over non-selective hand pulling and glyphosate treatments [14].

## Data Availability

The row data are available on request.

## Abbreviations

DAS: Days after sawing
No.: Number
FW: Fresh weight
DW: Dry weight
Wt: Weight
